# Navigation and perception of spatial layout in virtual echo-acoustic space

**DOI:** 10.1016/j.cognition.2020.104185

**Published:** 2020-04

**Authors:** C. Dodsworth, L.J. Norman, L. Thaler

**Affiliations:** Department of Psychology, Durham University, Durham DH1 3LE, UK

**Keywords:** Audition, Blindness, Sonar, Training, Learning, Neuroplasticity

## Abstract

Successful navigation involves finding the way, planning routes, and avoiding collisions. Whilst previous research has shown that people can navigate using non-visual cues, it is not clear to what degree learned non-visual navigational abilities generalise to ‘new’ environments. Furthermore, the ability to successfully avoid collisions has not been investigated separately from the ability to perceive spatial layout or to orient oneself in space. Here, we address these important questions using a virtual echolocation paradigm in sighted people. Fourteen sighted blindfolded participants completed 20 virtual navigation training sessions over the course of 10 weeks. In separate sessions, before and after training, we also tested their ability to perceive the spatial layout of virtual echo-acoustic space. Furthermore, three blind echolocation experts completed the tasks without training, thus validating our virtual echo-acoustic paradigm. We found that over the course of 10 weeks sighted people became better at navigating, i.e. they reduced collisions and time needed to complete the route, and increased success rates. This also generalised to ‘new’ (i.e. untrained) virtual spaces. In addition, after training, their ability to judge spatial layout was better than before training. The data suggest that participants acquired a ‘true’ sensory driven navigational ability using echo-acoustics. In addition, we show that people not only developed navigational skills related to avoidance of collisions and finding safe passage, but also processes related to spatial perception and orienting. In sum, our results provide strong support for the idea that navigation is a skill which people can achieve via various modalities, here: echolocation.

## 1 Introduction

Successful navigation involves finding the way, planning routes, and avoiding collisions in the environment (Moffat, 2009; Wolbers & Hegarty, 2010). Sighted people typically use vision to navigate their environment and this has been investigated using visual paradigms (Ekstrom, 2015). Navigational abilities, however, have also been demonstrated using non-visual paradigms, suggesting that navigation may be a cross-modal ability (Chebat, Maidenbaum, & Amedi, 2015; Kupers, Chebat, Madsen, Paulson, & Ptito, 2010; Levy-Tzedek, Maidenbaum, Amedi, & Lackner, 2016; Massiceti, Hicks, & van Rheede, 2018). Yet, there are important open questions.

People's ability to navigate and recognise routes using non-visual modalities has, for example, been investigated in blind and blindfolded sighted people using the Tongue Display Unit (TDU) (Kupers et al., 2010). Both blind and blindfolded sighted participants were able to use the TDU to navigate and recognise routes (Kupers et al., 2010), but navigation ability was always assessed within the same environments, so there is a possibility that participants may have acquired ‘stereotyped’ responses which would enable participants to complete the virtual routes without learning any sensory-driven navigational skills. Furthermore, the way in which participants perceived the environment was from an aerial perspective, as opposed to the first-person perspective that is experienced when navigating in real-life environments. Further research has investigated non-visual navigation from a first person perspective using the virtual EyeCane which uses sound to signal distances of surfaces in the environment, with a higher frequency of beeps transmitted to the user the closer a surface is (Levy-Tzedek et al., 2016). Another study investigated first-person perspective navigation using sound in a virtual environment (Massiceti et al., 2018). Participants wore a head mounted VR device which also tracked their orientation and head position, allowing them to complete the task by physically walking. Two acoustic methods were used. One was modeled on echolocation; the device emitted 900 ‘sound particles’ which produced a ‘pop’ upon colliding with virtual objects and then reflected back to the participant. The other method used source sound (i.e. a humming sound) to indicate the distance of approaching objects. Whilst both these studies (Levy-Tzedek et al., 2016; Massiceti et al., 2018) clearly show that acoustic information could be used for navigation, people took part in a maximum of two sessions so it is not clear to what degree navigational abilities might develop over time, or generalise to other, novel spaces. Furthermore, people's ability to avoid collisions and find their way was not assessed independently from their ability to perceive space or spatial layout.

In sum, whilst previous research has shown that people are able to improve their ability to find their way using non-visual cues, it is not clear to what degree learned abilities generalise to ‘new’ environments. Furthermore, the ability to successfully avoid collisions has not been investigated separately from the ability to perceive spatial layout or to orient oneself in space. Importantly, these processes are treated separately in the context of visual navigation. If a similar division of functions could be observed in non-visual navigation it would strongly support the idea of navigation truly being a cross-modal function. Here, we address these questions using a virtual echolocation paradigm in sighted people.

Echolocation allows for the perception of the environment through the reflection of sound (Jones, 2005). To echolocate, an organism emits a sound and uses the returning echoes to obtain information about the environment. Echolocation is predominantly used by non-human species, such as bats and dolphins (Griffin, 1958; Jones, 2005), but some blind humans have also developed this ability. Importantly, research on human echolocation suggests that it provides sensory benefits for blind individuals, as they are able to avoid obstacles (Kolarik et al., 2016, Kolarik et al., 2017; Supa, Cotzin, & Dallenbach, 1944; Worchel, Mauney, & Andrew, 1950) and obtain information about their environment, such as the distance, size, shape and material of objects (Hausfeld, Power, Gorta, & Harris, 1982; Kellogg, 1962; Milne, Goodale, & Thaler, 2014; Rice & Feintein, 1965. Reviews: Kolarik, Cirstea, Pardhan, & Moore, 2014; Thaler & Goodale, 2016). This ability to gain knowledge of the environment is an important element of navigation (Gillner & Mallot, 1998; Merrill, Yang, Roskos, & Steele, 2016).

The current study will use echolocation, in virtual echo-acoustic space, to investigate spatial navigation. Echolocation is acoustically complex. For example, the emission going out into space is not a uniform spread of sound waves, but there is a directional pattern of frequency and intensity forming a directional emission (Thaler et al., 2017). Furthermore, any emission not only leads to direct reflections from the environment to the echolocators ears (i.e. mirror-reflections), but there are also frequency dependent edge diffraction effects affecting echo spectrum and intensity (e.g. see Norman & Thaler, 2018), as well as higher order echoes and interactions across echoes. As a consequence, previous approaches to modelling echolocation in virtual spaces have either used simplified emission models, e.g. a cone of certain angular extent spreading sound equally within that cone (Massiceti et al., 2018), and/or simplified environments containing single or double reflectors in otherwise anechoic spaces (e.g. Schörnich, Nagy, & Wiegrebe, 2012; Wallmeier, Geßele, & Wiegrebe, 2013), and/or simplified reflection models (Massiceti et al., 2018). One way to get around acoustic inaccuracies is to measure binaural room impulse responses (BRIRs) with a real human or with an anthropometric manikin in a real space and to apply these to create a virtual echo-acoustic space (e.g. Flanagin et al., 2017; Wallmeier & Wiegrebe, 2014). Another way to do this is to make actual binaural recordings of emissions and echoes in specific locations and orientations in real space and to play these back to users when they traverse locations and orientation in respective virtual space. The latter approach is the approach we took for the current study. Thus, in our study participants navigated a virtual space constructed of an array of binaural recordings.

Fourteen sighted participants completed 20 virtual navigation training sessions over the course of 10 weeks. A key feature of the virtual navigation task was that participants learned to navigate using a specific set of virtual environments in sessions 1–18, whilst in sessions 19 and 20 they completed both trained (‘old’) and untrained (‘new’) environments. This allowed us to examine if people had learned a ‘stereotyped’ response, or if their skill in navigating using echo-acoustic information generalised to novel virtual spaces and thus represented ‘true’ sensory driven navigational ability. In addition, the ability to perceive the spatial layout of various environments was also assessed before and after training. Running this additional perceptual task, in addition to the virtual navigation task, enabled us to disentangle navigational processes related to avoidance of collisions and finding safe passage from processes related to spatial perception and orienting. Three blind people with experience in click-based echolocation also took part in single sessions of each task, i.e. without any training.

## 2 Materials & methods

### 2.1 Participants

Fourteen sighted echolocation beginners (8 males) aged 21–71 years (21, 21, 22, 22, 23, 24, 25, 27, 32, 35, 38, 48, 60, and 71) participated (M = 33.5, SD = 15.8, median = 26). All reported to have no prior echolocation experience and normal or corrected to normal vision. They all reported to have normal hearing and had normal hearing appropriate for their age group (ISO 7029:2017) assessed using pure tone audiometry (0.25–8 kHz).

To validate our paradigm, we also tested three people with experience in echolocation (EE1–EE3). EE1 was male and 50 years old at time of testing and totally blind since age 13 months due to enucleation. He has used echolocation as long as he can remember and uses it daily. EE2 was male and 35 years old at time of testing. He has bright light perception, and lost sight gradually from birth due to glaucoma. He has used echolocation since age 12 and uses it daily. EE3 was male and 24 years old at time of testing. He is totally blind. He lost his vision suddenly at age 12, due to unknown causes. At age 19 both eyes were removed to alleviate ocular discomfort. He has used echolocation since age 12, and uses it daily. These individuals did not take part in any training, but performed each experimental task once.

### 2.2 Virtual navigation task

To create a computer based virtual navigation task, three physical mazes were constructed. Sound recordings were made within these mazes, and then used to populate six virtual mazes (i.e. three original and three mirror versions). The virtual mazes were navigated by sighted blindfolded participants across 20 sessions, with two sessions per week spread over a 10-week period. The aim of the task was to train participants to navigate using echo-acoustic information. Importantly, a key feature of the task was that participants learned to navigate using a specific set of virtual environments in sessions 1–18. In sessions 19 and 20, we then examined how performance changed upon presentation of ‘old’ (trained) and ‘new’ (untrained) environments.

Echolocation experts did a single session of this task, without any training. The purpose was to validate the paradigm.

#### 2.2.1 Sound recording & editing

Sound recordings were made in a sound-insulated and echo-acoustic dampened room (approx. 2.9 m × 4.2 m × 4.9 m) lined with foam wedges (cut-off frequency 315 Hz). A 300 × 300 cm grid was mapped onto the floor of the anechoic chamber and subdivided into 25 cm^2^ squares. Three physical mazes were created using poster boards, with each individual panel measuring 90 × 90 cm, reaching up to 200 cm and its height centred on the manikin used during recording (see below).

**Fig. 1:**
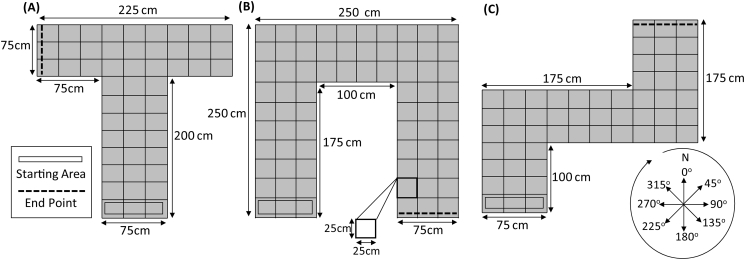
An illustration of (A) T-maze, (B) U-maze and (C) Z-maze and recording positions. In all diagrams, the box represents the starting area and the dashed black line symbolises the end point which was made from corrugated plastic sheets. Eight sound recordings (0°–315°) were made at each intersection in each route. All diagrams contain dimensions of each physical route.

Eight sound recordings were made in a clockwise direction at 0°, 45°, 90°, 135°, 180°, 225°, 270°, and 315° at each intersection point of the grid within each maze, with a north facing start point (Fig. 1). A speaker (Visaton SC5.9 ND, Visaton, Germany) mounted in front of the mouth of a manikin was used to play click-sounds (see Norman & Thaler, 2018 for anthropometric measurements of the manikin). The speaker was driven by a laptop (Dell Studio 1558; Intel i3 CPU 2.27 GHz; 4 GB RAM; Windows 7 pro 64 bit), external sound card (Creative Sound Blaster External Sound Card Model SB1240; Creative Technology Ltd., Creative Labs Ireland, Dublin, Ireland 24 bit; 96 kHz) and amplifier (Kramer 900 N; Kramer Electronics Ltd., Jerusalem, Israel). Sounds were produced by Audacity 2.0.2, with clicks modeled based on Thaler et al. (2017, PloS Comp Biology). The clicks and returning echoes were recorded by microphones (DPA SMK-SC4060 miniature microphones; DPA microphones, Denmark), placed inside the manikin's ears, connected to a PC via USB audio interface (Scarlett Focusrite 2i2; Focusrite PLC; High Wycombe, Bucks HP12 3FX, UK; 24 bit and 96 kHz). Computers and amplifier were located in a different room and connected through a panel.

**Fig. 2:**
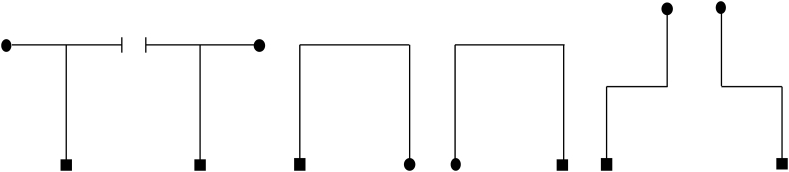
Line drawings illustrating the six mazes used to train or test echolocation ability. The square represents the starting position and the circle denotes the goal position within each route.

We recorded a T-maze, U-maze and Z-maze. Detailed information about each maze is presented in Fig. 1. The end point of each maze was acoustically differentiated by creating this wall using corrugated plastic sheets. The entrance of each maze was acoustically differentiated by having it open and facing a foam wall of the lab. The remaining walls – those that formed the exterior geometry – were constructed using poster boards. The recorded sounds were processed using MATLAB R2012b (The Mathworks, Natick, MA). The result was a single emission and returning echo for each position and angular orientation within a maze. To create mirror images of each maze, channels and locations were reassigned. This resulted in six distinct virtual mazes (Fig. 2).

#### 2.2.2 Set up & computer program

MATLAB R2018b (The Mathworks, Natick, MA) and modified functions from the Psychtoolbox library (Brainard, 1997) were used to run the experiment on a laptop (Dell Latitude E7470; Intel Core i56300U CPU 2.40; 8 GB RAM; 64-bit Windows 7 Enterprise) with external sound card (Creative Sound Blaster External Sound Card Model SB1240; Creative Technology Ltd., Creative Labs Ireland, Dublin, Ireland; 24 bit and 96 kHz). Echolocation stimuli were presented through headphones (Etymotic ER4B; Etymotic Research, Illinois, USA), at a level at which the highest intensity sound was approximately 80 dB SPL.

To navigate, participants used the computer keyboard. Pressing any key would start a trial. Each press of the ‘W’ key would move the participant one step forward in the virtual maze and the ‘S’ key would move them one step backwards, but still facing in the same direction. Each press of the ‘A’ key would rotate the participant 45° in an anti-clockwise direction and the ‘D’ key would rotate them 45° clockwise. When participants pressed a key to update their position/orientation in the maze, a new sound recording of the click-sound at that position/orientation would begin to play. This sound would repeat at a rate of one click per second until participants pressed a key to update their position/orientation. If participants pressed a key that would result in them colliding with a virtual wall, an error tone would be presented and participants would remain in the same virtual position. In each trial, participants had 180 s to locate and move to the end point of the maze (i.e. the wall made of corrugated plastic), at which point the trial would end. Auditory feedback was used at the end of each trial to indicate to participants whether they were successful or not. Participants would then begin the next trial after pressing a key.

#### 2.2.3 Procedure

All sighted participants were asked to wear a blindfold and close their eyes when completing the virtual navigation task. Each participant was assigned to one of two groups, which differed only in the set of mazes that they trained with. One group started with the T-maze (left turn), U-maze (right-right turn) and Z-maze (right-left turn), and the other started with the mirror version of each maze. At the beginning of the first session, a demo trial was completed in order to gain familiarity with the controls and the task. All sighted participants completed 20 sessions in total. Sessions 1–18 consisted of 18 trials each, with each of the three mazes being presented six times in an unpredictable order. Sessions 19 and 20 contained 36 trials – 18 of which contained mazes that had been previously navigated, and the remaining 18 of which contained mazes that were ‘new’ (i.e. the three mirror versions that participants had not trained with in sessions 1–18).

Participants could enter each maze at one of four positions in front of the starting wall in sessions 1–14, and they always faced straight into the maze (i.e. at 0°). From session 15 onward, participants' starting orientation was unpredictable – they could be facing one of 3 orientations (0°, 45°, or 315°) meaning there were 12 different possible starting conditions. All starting locations and orientations were randomised and participants were not informed. In addition to this, from session 15 onward participants would receive a 15 s time-out if they made a collision with one of the virtual walls, during which time they would not hear any echolocation stimuli and would be unable to change position/orientation in the maze. These changes implemented from session 15 onward were introduced in order to encourage participants to use an effective stimulus-driven navigation strategy.

Expert echolocators completed a single session of 36 trials. The session was equivalent in all other aspects to any of the sessions 1–14 in sighted participants. The echolocation expert who still had eyes (EE2) wore a blindfold.

### 2.3 Perception of spatial layout task

Spatial perception abilities in sighted participants were assessed before and after training. A computer program led participants through each maze, as well as through a selection of control conditions (see subsequent sections for more details). The task was to determine which turns the route took or if a control sound was played. Running this task allowed us to measure people's ability to use echoic sounds to determine spatial features of the environment.

Echolocation experts completed a single session of this task, without any training. The purpose was to validate the paradigm.

#### 2.3.1 Sound recording & editing

The same sounds used in the virtual navigation task (2.2.1) were used for the perception of spatial layout task.

For each of the six mazes, we created two samples by selecting recordings corresponding to a specific sequence of locations and orientations within that maze. The resulting sound files were 10.53 s in length and contained 18 clicks and echoes, each separated by 600 ms. There were 12 sound files in total, which were assigned to one of three categories: (1) single turn route, (2), two turn route with both turns going into the same direction, (3), two turn route with both turns going into different directions. In addition to these spatially coherent route sounds, there were also two types of control sounds: scrambled route sounds and clicks with no echoes. Scrambled route sounds were created for each of the six routes in order to create sound files that had exactly the same acoustic information (i.e. timing, clicks and echoes), but did not convey spatially coherent information. To do this, the individual click-echo sounds in each route sound file were randomly shuffled and pieced together so that there was no coherent route. In order to create a secondary set of control stimuli (i.e. stimuli with clicks but not containing any echoes), a sound recording was used during which the manikin had been placed facing the foam padded wall in the anechoic chamber. The sound was then repeated at the same temporal sequence as sounds in ‘route’ and ‘scrambled’ sound files.

In total, five types of sound stimuli were created: single-turn route, two-turns-same route, two-turns-different route, scrambled route, and clicks with no echoes. Due to the way in which stimuli were created, stimuli containing echoes (‘route’ and ‘scrambled’ stimuli) were of higher RMS intensity than stimuli not containing echoes (‘clicks’) (T and T-scrambled: −41.4 dB; U and U scrambled: −41.4 dB; Z and Z-scrambled: −40.8 dB; Clicks: −44.2 dB).

#### 2.3.2 Set up & computer program

A laptop (Dell Latitude E7470; Intel Core i56300U CPU 2.40; 8 GB RAM; 64-bit Windows 7 Enterprise) and MATLAB R2018b (The Mathworks, Natick, MA) were used to run the spatial layout perception task. All audio stimuli were presented via an external sound card (Creative Sound Blaster External Sound Card Model SB1240; Creative Technology Ltd., Creative Labs Ireland, Dublin, Ireland; 24 bit and 96 kHz) and amplifier (Kramer 900 N; Kramer Electronics Ltd., Jerusalem, Israel) over insert earphones (Model S14, Sensimetrics, Malden, MA, USA) at about 80 dB SPL. Audio stimuli had been equalised for the non-flat frequency response of the headphones using filters provided by the manufacturer. There were 30 trials per block, in which each of the five types of stimuli were presented 6 times in a random order.

#### 2.3.3 Procedure

Participants completed one session before and one after training with the virtual navigation task. Each session contained two blocks of 30 trials each. Participants were asked to wear a blindfold and close their eyes. Initially, participants were presented twice with an example of each type of sound and received feedback. If they wanted to hear the sounds again, this was repeated. When experimental trials commenced, participants were asked to respond verbally as to which type of sound they had heard. They could give one of 5 responses – single-turn route, two-turns-same route, two-turns-different route, scrambled route, and clicks with no echoes. Their response was recorded by the experimenter and no feedback was given. The next trial followed immediately. Each session took a maximum of 20 min to complete.

Echolocation experts completed a single session without any training. The session was equivalent to any session in sighted participants. The echolocation expert who still had eyes (EE2) wore a blindfold.

## 3 Results

### 3.1 Virtual navigation task

We first examined how performance changed across 18 training sessions when multiple mazes were repeatedly presented to sighted participants. We measured the time taken to complete each maze, the number of collisions made (i.e. bumping into virtual walls), and the proportion of mazes successfully completed in each of the 18 training sessions.

In addition, to describe how participants moved around in the space and how this changed with training, we calculated the difference in time participants spent within each square section of the maze during sessions 13 and 14 (these were the final sessions before time-outs and randomised starting orientation were introduced) compared to sessions 1 and 2.

We then examined how well participants were able to navigate ‘old’ (trained) and ‘new’ (untrained) mazes in sessions 19 and 20. The logic here was to determine if people had learned a ‘stereotyped’ response or if their skill in navigating using echo-acoustic information generalised to novel virtual spaces. Again, we measured the time taken to complete each maze, the number of collisions made and the proportion of mazes successfully completed.

#### 3.1.1 Performance across repeated training sessions

We ran repeated measures ANOVAs to examine the effect of sessions 1–14 and sessions 15–18 on the time taken to navigate, the number of collisions made and the proportion of routes successfully completed. This subdivision is because in session 15, participants had been introduced to more difficult starting positions and a 15 s time-out when a collision was made. We also ran a paired sample *t*-test to compare performance in sessions 14 and 15 for the time taken to navigate, the number of collisions made and the proportion of mazes successfully completed. When sphericity could not be assumed we applied Greenhouse-Geisser correction to F-Ratios (F_GG_).

Data for each individual echolocation expert were compared to data from sighted participants in session 14 using *t*-tests adapted for single case designs (Crawford & Garthwaite, 2002; Crawford & Howell, 1998). This *t*-test considers the sample of sighted participants the ‘control sample’ and data of an individual echolocator a ‘single case’ to which the control sample is compared. Importantly, in calculating the test statistic the score of the individual is considered a sample with its own sampling error. Furthermore, we compared the group of expert echolocators (*n* = 3) to the group of sighted participants (*n* = 14). For this we used both independent samples *t*-tests, as well as non-parametric Mann-Whitney *U* tests, because of different sample sizes in the two groups (i.e. 3 vs. 14).

##### 3.1.1.1 Time taken to complete maze

**Fig. 3:**
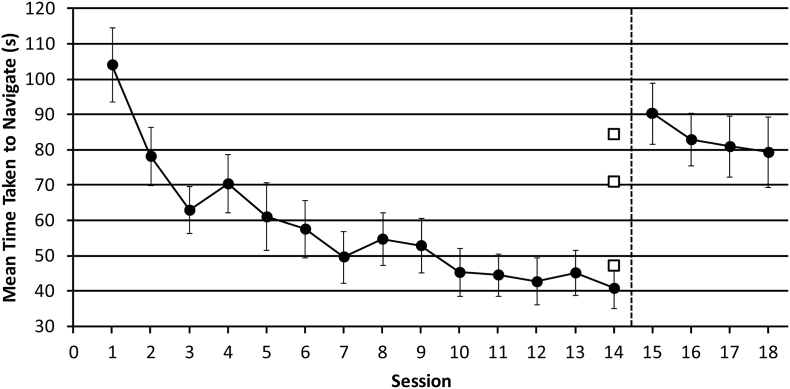
The mean time taken (seconds) to complete various mazes in sessions 1–18. In session 15, unpredictable starting orientations were introduced, along with a 15 s time-out when a collision occurred. This is represented by the dashed black line. Data from sighted participants are shown as solid circles, with error bars representing the standard error of the mean. Data from experts who completed only a single session are shown as open squares. For comparison, they have been plotted at session 14 (i.e. after sighted participants have had 13 sessions of practice with the task that experts did in a single session without training).

When examining the time taken to complete each maze, we found a significant effect of ‘session’ (F_GG(3.005, 39.066)_ = 20.926, *p* < .001, η^2^ = 0.617), and a significant linear trend (F_(1,13)_ = 41.189, *p* < .001, η^2^ = 0.760). Taken with Fig. 3, this shows that the average time taken to complete each maze significantly decreased as sessions progressed from 1 to 14. When comparing performance in sessions 14 and 15, we found a significant difference (t_(13)_ = 5.789, *p* < .001), with participants completing each maze in less time in session 14 (M = 40.866) compared to session 15 (M = 90.329). The difference in the time taken to navigate between sessions 14 and 15 may be due to the 15 s time-out which was imposed when each collision was made in session 15. On average, participants made 2 collisions in session 15 (see Section 3.1.1.2) which would result in an additional time of 30 s to complete each maze compared to session 14. Note, however, that participants did in fact require, on average, an additional time of almost 50 s, not 30 s, to complete each maze, suggesting that other factors play a role, too. For example, the additional 20 s might be due to the more difficult starting positions, which were also introduced in session 15, as well as participants possibly adopting a strategy that is more attentive to the echolocation sounds.

We found no significant difference in the average time taken to complete each maze in sessions 15–18 (F_(3,39)_ = 1.820, *p* = .160, η^2^ = 0.123).

To compare data from expert echolocators and sighted participants we used a *t*-test adapted for the comparison of single cases to a control sample (Crawford & Garthwaite, 2002; Crawford & Howell, 1998). We examined the average time to complete each maze and found that data from three expert echolocators did not significantly differ from data from sighted participants in session 14 (A: t_(13)_ = 1.948, *p* = .073, B: t_(13)_ = 0.014, *p* = .989, C: t_(13)_ = 1.346, *p* = .202).

When comparing data from the group of expert echolocators to the group of sighted participants the independent samples *t*-test revealed there was no significant difference (t_(15)_ = 1.971, *p* = .067) in the average time taken to complete each maze. Furthermore, we found the Mann-Whitney *U* test comparing mean ranks between expert echolocators and sighted participants was also non-significant (U = 8.00, z = −1.638, *p* = .121).

##### 3.1.1.2 Number of collisions made

**Fig. 4:**
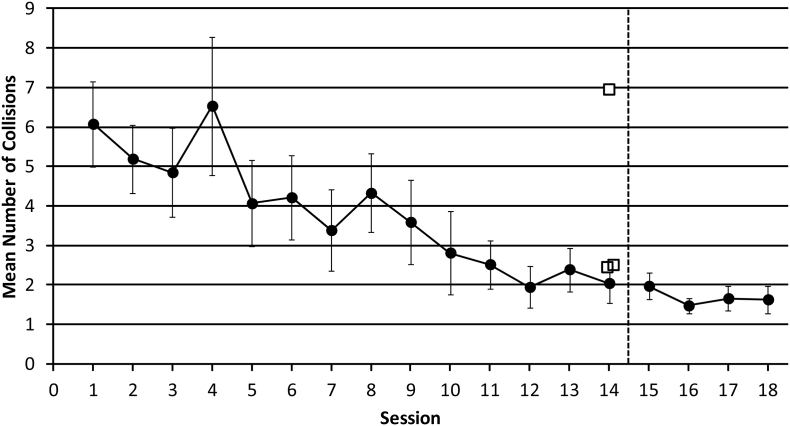
The mean number of collisions made in sessions 1–18. In session 15, unpredictable starting orientations were introduced, along with a 15 s time-out when a collision occurred. This is represented by the dashed black line. Data from sighted participants are shown as solid circles, with error bars representing the standard error of the mean. Data from experts who completed only a single session are shown as open squares. For comparison, they have been plotted at session 14 (i.e. after sighted participants have had 13 sessions of practice with the task that experts did in a single session without training).

When examining the number of collisions made in sessions 1–14, we found a significant effect of ‘session’ (F_GG(2.512, 32.657)_ = 5.779, *p* = .004, η^2^ = 0.308) and a significant negative linear trend (F_(1,13)_ = 27.848, *p* < .001, η^2^ = 0.682). Taken with Fig. 4, this shows that the average number of collisions decreased as training progressed from sessions 1–14.

We then compared the number of collisions made between sessions 14 and 15 and found no significant difference (t_(13)_ = 0.144, *p* = .888), with an average of 2.044 collisions in session 14 and 1.972 collisions in session 15. We also found no differences in the number of collisions made between sessions 15–18 (F_(3,39)_ = 2.336, *p* = .089, η^2^ = 0.152).

To compare data from expert echolocators and sighted participants we used a *t*-test adapted for the comparison of single cases to a control sample (Crawford & Garthwaite, 2002; Crawford & Howell, 1998). When we examined the number of collisions made, we found that data from two expert echolocators did not significantly differ from data from sighted participants in session 14 (A: t_(13)_ = 0.209, *p* = .084, B: t_(13)_ = 0.238, *p* = .815), but that data from one expert echolocator did differ significantly (C: t_(13)_ = 2.559, *p* = .024), with the number of collisions being significantly greater (M = 6.944) than that made by sighted participants in session 14 (M = 2.044).

The independent samples *t*-test revealed there was no significant difference (t_(2.149)_ = 1.145, *p* = .364) between the group of expert echolocators and sighted participants when considering the mean number of collisions made. Furthermore, the Mann-Whitney *U* test comparing mean ranks between expert echolocators and sighted participants was also non-significant (U = 8.00, z = −1.640, *p* = .121).

##### 3.1.1.3 Proportion of mazes successfully completed

**Fig. 5:**
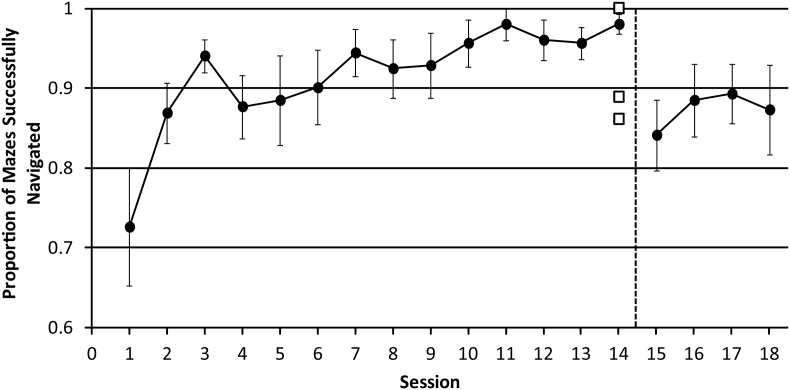
The proportion of mazes successfully navigated in sessions 1–18. In session 15, unpredictable starting orientations were introduced, along with a 15 s time-out when a collision occurred. This is represented by the dashed black line. Data from sighted participants are shown as solid circles, with error bars representing the standard error of the mean. Data from experts who completed only a single session are shown as open squares. For comparison, they have been plotted at session 14 (i.e. after sighted participants have had 13 sessions of practice with the task that experts did in a single session without training).

When examining the proportion of mazes successfully completed in sessions 1–14 we found a significant effect of ‘session’ (F_GG(2.578,33.517)_ = 6.995, *p* = .001, η^2^ = 0.350) and significant positive linear trend (F_(1,13)_ = 14.377, *p* = .002, η^2^ = 0.525). As Fig. 5 shows, participants became increasingly successful at navigating as sessions progressed. We also found a significant difference in performance between sessions 14 and 15 (t_(13)_ = 3.381, *p* = .005), with a greater proportion of mazes successfully completed in session 14 (M = 0.980), compared to session 15 (M = 0.841). This is likely due to the additional time-out and more difficult starting orientations from session 15 onwards, which could possibly make it more difficult to complete the mazes within the time limit. We also examined the effect of ‘session’ on the proportion of mazes successfully completed in sessions 15–18 and found no significant effect (F_GG(1.853, 24.087)_ = 0.995, *p* = .379, η^2^ = 0.071).

To compare data from expert echolocators and sighted participants we used a *t*-test adapted for the comparison of single cases to a control sample (Crawford & Garthwaite, 2002; Crawford & Howell, 1998). When examining the proportion of mazes successfully completed, we found that data from two expert echolocators did not significantly differ from sighted participants in session 14 (B: t_(13)_ = 0.411, *p* = .688, C: t_(13)_ = 1.871, *p* = .084). Data from one expert echolocator did significantly differ (A: t_(13)_ = 2.446, *p* = .029), with this echolocator completing significantly fewer mazes (M = 0.861) compared to sighted participants in session 14 (M = 0.980).

When comparing data from the group of expert echolocators to the group of sighted participants the independent samples *t*-test revealed there was no significant difference (t_(15)_ = −1.951, *p* = .070) between the group of expert echolocators and sighted participants when considering the proportion of mazes successfully completed. Furthermore, the Mann-Whitney *U* test comparing mean ranks between expert echolocators and sighted participants was also non-significant (U = 10.500, z = −1.643, *p* = .197).

##### 3.1.1.4 Time spent in sections of the maze

**Fig. 6:**
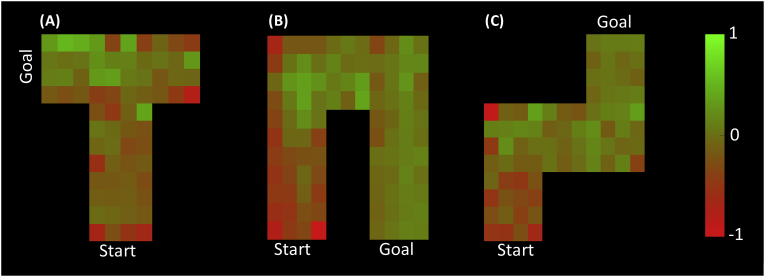
A heat map showing the relative time difference spent at each point in the T-maze (A), U-maze (B) and Z-maze (C) in sessions 13–14 compared to sessions 1–2. Greener points indicate relatively more time spent at that point in sessions 13–14 and redder points indicate relatively more time spent in the sessions 1–2. The units of the colormap are normalised time units, where 1 represents longest time spent in sessions 13–14 relative to sessions 1–2, and − 1 represents shortest time spent in sessions 13–14 relative to sessions 1–2.

To describe how participants moved around in the space and how this changed with training, we calculated the difference in time participants spent within each square section of the maze during sessions 13 and 14 (these were the final sessions before time-outs and randomised starting orientation were introduced) compared to sessions 1 and 2. This was done separately for each maze shape (T, U, Z) regardless of its orientation, so data from original and mirror versions of T, U and Z mazes were first flipped laterally such that spatial positions of start and goal areas corresponded across original and mirrored versions. For each participant, the mean time spent at each point across sessions 1 and 2 was calculated and normalised between 0 and 1. These data were then averaged across participants. The same was done for sessions 13 and 14. The mean normalised data from the first two sessions were then subtracted from the mean normalised data from the last two sessions. The result is a heat map image (Fig. 6) that shows the relative time difference spent at each point in the maze in sessions 13–14 compared to sessions 1–2. It is clear from the image that, with training, participants began to spend less time in the area of the maze close to the start and spend more time in the area close to the goal. It is also clear that, with practice, people learned to spend less time in certain corners of the mazes.

#### 3.1.2 Performance for ‘old’ and ‘new’ mazes

A paired sample *t*-test was used to compare performance for ‘old’ (trained) and ‘new’ (untrained) mazes in sessions 19 and 20. We examined the time taken to navigate, the number of collisions made and the proportion of mazes successfully completed.

**Fig. 7:**
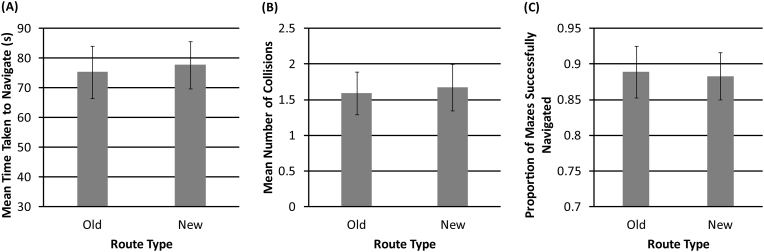
(A). Mean time taken (seconds) to navigate ‘old’ and ‘new’ mazes. (B). Mean number of collisions made when navigating ‘old’ and ‘new’ mazes. (C). Proportion of ‘old’ and ‘new’ mazes successfully navigated. Error bars represent the standard error of the mean across participants.

Overall, we found no significant differences between performance for ‘old’ mazes compared to ‘new’ mazes in sessions 19 and 20. Specifically, there was no significant difference (t_(13)_ = 0.968, *p* = .351) between the time taken to navigate ‘old’ (M = 75.313) and ‘new’ (M = 77.751) mazes. There was no significant difference (t_(13)_ = 1.020, *p* = .326) in the number of collisions when navigating ‘old’ (M = 1.591) and ‘new’ (M = 1.671) mazes. Similarly, there was no significant difference (t_(13)_ = 0.327, *p* = .749) in the proportion of mazes successfully completed for ‘old’ (M = 0.889) and ‘new’ (M = 0.883) mazes. This is shown in Fig. 7.

### 3.2 Perception of spatial layout task

To examine participants' ability to recognise spatial elements of routes, we compared the effect of ‘session’ (pre-training vs post-training). We calculated the proportion of correct responses given for echo identification, scrambled vs. route identification, stimulus identification and route identification.

When considering echo identification, a response was identified as correct when participants responded with ‘no echo’ when stimuli containing no echoes were present, and also when participants gave any other response when any of the other stimuli were presented (e.g. if a ‘single turn’ route was labelled as ‘scrambled’, then this would be classed as correct because the sound contains echoes). Thus, echo identification measures participants' ability to distinguish echo from non-echo sounds.

When considering scrambled vs. route identification, a response was identified as correct when participants gave a ‘scrambled’ response to a scrambled sound, but also when they gave any of the route responses when any of the route sounds were presented (regardless of whether it was a single turn, two-turn-same or two-turn-different). Thus, scrambled vs. route identification measures participants' ability to distinguish spatially coherent echo-acoustic sounds from spatially incoherent echo-acoustic sounds.

When considering stimulus identification, a response was identified as correct when the correct stimulus type (single turn; two-turns-same; two-turns-different; scrambled; no echo) was identified when it was presented. Thus, stimulus identification measures participants' ability to correctly identify specific echo-acoustic stimuli including non-echo and scrambled sounds.

When considering route identification, a response was correct when participants identified the specific route (single turn; two-turn-same; two-turn-different) when it was presented. Thus, route identification measures participants' ability to correctly identify specific echo-acoustic routes.

A significant effect of ‘session’ (t_(13)_ = 2.215, *p* = .045) was found when examining echo identification, with participants identifying a greater proportion of stimuli containing echoes after training (M = 1.00), compared to before training (M = 0.985). We also found a significant effect of ‘session’ (t_(13)_ = 5.422, *p* < .001) for scrambled vs. route identification, with participants better able to discriminate scrambled from coherent routes after training (M = 0.897), compared to before training (M = 0.773). When considering the ability to identify specific stimuli, we also found a significant effect of ‘session’ (t_(13)_ = 6.411, *p* < .001). A greater proportion of stimuli were correctly identified after training (M = 0.714), compared to before training (M = 0.532). We also found a significant effect of ‘session’ (t_(13)_ = 5.027, *p* < .001) when examining route identification. Participants were able to correctly identify a greater proportion of routes after training (M = 0.637), compared to before training (M = 0.426).

To compare data from expert echolocators and sighted participants in the ‘post’ training session we used a *t*-test adapted for the comparison of single cases to a control sample (Crawford & Garthwaite, 2002; Crawford & Howell, 1998). This *t*-test considers the sample of sighted participants as the ‘control sample’ and data of an individual echolocator as a ‘single case’ to which the control sample is compared. Importantly, in calculating the test statistic the score of the individual is considered a sample with its own sampling error. Furthermore, we compared the group of expert echolocators (*n* = 3) to the group of sighted participants (*n* = 14). For this we used both independent samples *t*-tests, as well as non-parametric Mann-Whitney *U* tests, because of different sample sizes in the two groups (i.e. 3 vs. 14).

**Fig. 8:**
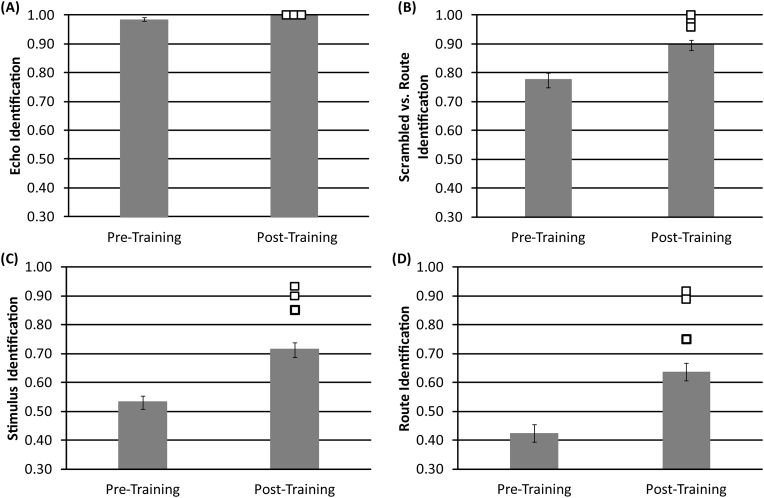
The proportion of correct responses given for (A) echo identification, (B) scrambled vs. route identification, (C) stimulus identification and (D) route identification. Data from sighted participants are shown as grey bars, with error bars representing the standard error of the mean across sighted participants. Data from experts who did only a single session without training are shown as open squares. For comparison, they have been plotted as ‘post’ training, i.e. after sighted participants have had 20 session of echo-acoustic training with the virtual navigation task.

For echo identification (Fig. 8A) sighted participants as well as experts all had a score of 1. When examining scrambled vs. route identification (Fig. 8B) , we found that data from three expert echolocators did not significantly differ from sighted participants data after training (A: t_(13)_ = 1.215, *p* = .246; B: t_(13)_ = 1.522, *p* = .152; C: t_(13)_ = 0.908, *p* = .381). Furthermore, the independent samples *t*-test revealed there was no significant difference (t_(15)_ = 2.119, *p* = .051) between the group of expert echolocators and sighted participants. However, the Mann-Whitney U test comparing mean ranks between expert echolocators and sighted participants was significant (U = 4.500, z = −2.090, *p* = .032) with expert echolocators better able to discriminate between scrambled and coherent routes (M = 0.979), compared to sighted participants (M = 0.896).

When examining stimulus identification (Fig. 8C), we found that data from two expert echolocators did not differ significantly from sighted participants data in the post-training session (B: t_(13)_ = 1.351, *p* = .200; C: t_(13)_ = 1.843, *p* = .082), but data from one expert echolocator did significantly differ (A: t_(13)_ = 2.169, *p* = .049), with a greater proportion of stimuli being correctly identified (M = 0.933) as compared to sighted participants after training (M = 0.713). When comparing data from the group of expert echolocators to the group of sighted participants the independent samples *t*-test revealed a significant difference (t_(15)_ = 3.084, *p* = .008), with a greater proportion of stimuli identified by expert echolocators (M = 0.894) than sighted participants (M = 0.714). Furthermore, the Mann-Whitney *U* test comparing mean ranks between expert echolocators and sighted participants was also significant (U = 1.500, z = −2.464, *p* = .006).

When examining route identification (Fig. 8D), we found that data from two expert echolocators did not significantly differ from sighted participants in the post-training session (B: t_(13)_ = 0.966, *p* = .352; C: t_(13)_ = 2.154, *p* = .051), but data from one expert echolocator did significantly differ (A: t_(13)_ = 2.394, *p* = .032), with the expert echolocator correctly identifying a greater proportion of routes (A: M = 0.917), compared to sighted participants after training (M = 0.637). When comparing data from the group of expert echolocators to the group of sighted participants, the independent samples *t*-test revealed a significant difference (t_(15)_ = 3.069, *p* = .008), with expert echolocators correctly identifying a greater proportion of routes (M = 0.852) compared to sighted participants after training (M = 0.637). Furthermore, the Mann-Whitney U test comparing mean ranks between expert echolocators and sighted participants was also significant (U = 2.500, z = −2.351, *p* = .012).

### 3.3 Relationship between participant performance and age

The age range in our sample of participants was large. To investigate if age was related to performance we conducted correlation analyses. Specifically, we correlated sighted participants' age with their score on each of our outcome measures, i.e. performance in the virtual navigation task (time taken to complete the maze, collisions and proportion of successes in sessions 14, 15 and 18, along with performance in navigating ‘old’ (untrained) and ‘new’ (trained) mazes in sessions 19 and 20) and performance in the spatial layout task (pre vs post for echo identification, route vs. scrambled identification, stimulus identification and route identification). The only significant correlation was between age and the proportion of mazes successfully completed in the virtual navigation task in session 14 (r_(12)_ = −0.547; *p* = .043). However, this was driven by a single participant, and when we removed this participant the correlation became non-significant (r_(12)_ = 0.227; *p* = .457). It is possible that we might observe significant correlations between age and performance with a larger sample size, but at present our data do not support the conclusion that age is related to any of our outcome measures.

## 4 Discussion

Here, we show that sighted people can learn to use echo-acoustic cues to navigate virtual space. An improvement in performance was observed across the 10-week training period, i.e. reduction in the time taken to navigate, fewer collisions and an increased completion rate of virtual mazes. Furthermore, following training, we found no difference in performance when navigating ‘old’ (trained) and ‘new’ (untrained) mazes. This transfer of knowledge suggests participants were learning and utilising sensory driven navigational skills, and not just executing automated responses. When considering the ability to perceive the spatial layout of virtual routes, participants were able to identify all types of stimuli more accurately after training, showing an improvement in the ability to identify and judge the spatial layout of various environments. It is important to note that participants were very good at detecting echoes even before training, so that the ability to identify the absence and presence of echoes does not imply the ability to perceive the spatial layout of the environment.

Three expert echolocators also performed the experimental tasks without any training. Whilst one echolocator performed significantly worse for number of collisions compared to sighted trained participants, and one for number of successes, overall experts performed at a very high level without any training, suggesting that they were able to apply their echo-acoustic knowledge based on experience in real space to our virtual space. Upon questioning, the echolocators did comment on the high quality of the acoustics and that it sounded spatially meaningful, but they also pointed out that it sounded different from the way they usually hear echoes through their own ears and using their own clicks. Thus, whilst overall expert performance validates our paradigm, and confirms that the tasks require echo-acoustic processing, it also demonstrates that a certain adaptation might be required for people who are experienced in the use of click-based echolocation.

Importantly, by training and assessing abilities in different environments, we have shown participants are learning a sensory driven skill, as opposed to a ‘stereotyped response’. This is an important extension to previous work (i.e. Kupers et al., 2010;Levy-Tzedek et al., 2016; Massiceti et al., 2018). Furthermore, by using both an ‘active’ navigation task requiring participants to find their way, as well as a ‘passive’ spatial perceptual task (which was not part of the training), we have also shown that participants learned two different aspects of echo-acoustic navigation. The first is the ability to use sound echoes to avoid collisions and to find safe passage, highlighted by the improvement in successful route completion within the time limit, reduction of collisions and reduction in navigation time. The second is the ability to judge the spatial layout of environments, shown by the improvement in ability to perceive and judge characteristics of various routes after training. Research in the visual domain suggests a similar division of skill. Vision provides information which allows people to complete a route without collision (Hildreth, Beusmans, Boer, & Royden, 2000; Wallis, Chatziastros, Tresillian, & Tomasevic, 2007) and gain knowledge of spatial relations within an environment (Gillner & Mallot, 1998). In sum, our results provide strong support for the idea that navigation is a skill which can be achieved via various modalities.

The ability to utilise echo-acoustic cues to navigate improved considerably over the 10-week training period, but participants were not able to navigate without making a collision or successfully complete all routes within the time limit. Despite this, participants in the current experiment were able to navigate virtual mazes faster than those in previous studies (Levy-Tzedek et al., 2016; Massiceti et al., 2018), along with successfully completing more mazes within the allocated time (Kupers et al., 2010). However, when considering the number of collisions made, previous research is quite varied. In the current experiment, participants, on average, collided with a wall twice within each maze, whereas previous research varies from 1.3 collisions up to 15 collisions using various acoustic methods (Levy-Tzedek et al., 2016; Massiceti et al., 2018). Similarly, all our sighted participants also improved in their ability to identify the spatial layout of various environments, but even though they showed vast improvements, they still made some errors. Yet, even echolocation experts did not perform perfectly in our experimental tasks, suggesting that our paradigm was intrinsically difficult.

In addition to the above, it may be possible that the tasks in the current experiment were more difficult than those of previous experiments due to the reliance on echo-acoustic cues only. In previous experiments, sighted participants had some form of visual exposure (Levy-Tzedek et al., 2016; Massiceti et al., 2018). For example, when learning to use the virtual EyeCane, participants received both visual and auditory information (Levy-Tzedek et al., 2016) and despite there being no overlap between the spaces used to demonstrate the EyeCane to participants, and those used during the experiment, it does not rule out the possibility that participants benefitted from having had some form of correspondence between visual and auditory information. Similarly, Massiceti et al. (2018) asked participants to complete visual conditions as well as auditory conditions. Whilst there were no visual-audio trials, the visual exposure may mean that participants obtained spatial information regarding the layout of the environment, for example what corners looked like, what the obstacles looked like and what step sizes were like. In contrast, participants in the current experiment did not receive any visual information related to the routes, which could have resulted in the task being more difficult. Despite this, the lack of visual information means we were able to simulate how spatial information would be acquired when vision was not available, e.g. by people who are blind.

The paradigm we have developed here is the first ‘acoustically correct’ virtual echo-acoustic navigation task. We made binaural recordings of emissions and echoes in real space which were then replayed to participants when navigating the corresponding virtual space. The considerable improvement in navigation ability, over the 10-week training period, highlights this paradigm as a feasible way to test navigation using echolocation. Going forward, it may be beneficial to consider creating more complex environments to accurately represent everyday navigational situations and provide a more comprehensive test of navigation abilities using echolocation. This could be as simple as including corridors of different widths and lengths or could involve introducing multiple rooms containing relevant objects. To further improve this paradigm, it may also be beneficial to allow people the opportunity to adjust the click intensity when completing the navigation based tasks. Currently, recording and playback was at a fixed intensity, but it has been shown that expert echolocators dynamically adjust their clicks pending the situation, for example they may make their clicks louder for weaker echoes or increase number of clicks made (Thaler et al., 2018; Thaler et al., 2019). Allowing participants to control the intensity of clicks may make this paradigm more representative of everyday situations.

Previous research has repeatedly shown that echolocation is a useful skill for blind people (for reviews see Kolarik et al., 2014; Thaler & Goodale, 2016). It has been reported that using echolocation can lead to enhanced mobility in unfamiliar places (Thaler, 2013), possibly allowing blind people a higher degree of independence in everyday life. The paradigm introduced here could be a useful training tool for visually impaired people as virtual environments can be controlled in their complexity (Karimpur & Hamburder, 2016; Van der Ham, Faber, Venselaar, Kreveld, & Löffler, 2015) and adjusted to reflect real life environments. Importantly, virtual environments offer the opportunity to explore an unknown environment without the risk of injury (Manduchi & Kurniawan, 2011; Templeman, Denbrook, & Sibert, 1999). Yet, it is unclear if the virtual echolocation navigation skills gained in our paradigm would transfer to a real life environment in which participants would physically navigate using echolocation. The echolocation experts performed at a very high level without any training, suggesting that they were able to transfer echo-acoustic knowledge gained in the real world to our virtual space. However, the transfer from skills acquired in virtual space into real space might be a different matter. Whilst previous research has investigated the issue of transfer of spatial knowledge from virtual to real environments, it has not been within the context of echolocation. For example, navigation performance within a visually presented virtual reality mobile based app was significantly correlated with performance in a real life wayfinding task (Coutrot et al., 2019), suggesting that virtual-real transfer is possible. Furthermore, using spatial information from an audio based virtual environment, blind people were able to successfully navigate a real world building (Connors, Chrastil, Sánchez, & Merabet, 2014; Merabet, Connors, Halko, & Sánchez, 2012). In sum, whilst previous research strongly suggests the possibility of transfer from virtual space to the real world, future work is required to test how this applies in the context of echolocation.

## Funding sources

This work was supported by a grant from the Biotechnology and Biological Sciences Research Council United Kingdom to LT (BB/M007847/1). The funders had no role in study design, data collection and analysis, decision to publish, or preparation of the manuscript.

## Author contribution statement

LT: Conceptualization; CD: Data curation; CD: Formal analysis; LT: Funding acquisition; CD: Investigation; LN, LT, CD: Methodology; LT: Project administration; LN, CD, LT: Resources; LN: Software; LT: Supervision; CD, LT: Validation; CD, LN: Visualization; CD, LT: writing original draft; LN, LT, CD: review & editing.

## Appendix A Supplementary data

Excel file containing data reported in the results section, broken down for each individual participant. Image 1
